# Protective effects of 1,25 dihydroxyvitamin D_3_ and its analogs on ultraviolet radiation-induced oxidative stress: a review

**DOI:** 10.1080/13510002.2020.1731261

**Published:** 2020-02-25

**Authors:** Shemani Vishalya Jagoda, Katie Marie Dixon

**Affiliations:** Discipline of Anatomy and Histology, School of Medical Sciences, Faculty of Medicine and Health, The University of Sydney, Camperdown, Australia

**Keywords:** Vitamin D, 1,25-dihydroxyvitamin D3 (1,25D), oxidative stress, reactive oxygen species (ROS), p53, metallothionein (MT), DNA damage, c-Jun N-terminal kinases (JNK)

## Abstract

The active vitamin D compound, 1,25-dihydroxyvitamin D3 (1,25D) is produced in skin cells following exposure to ultraviolet radiation (UV) from the sun. However, there are many harmful effects of UV which include DNA damage caused by direct absorption of UV, as well as that caused indirectly via UV-induced reactive oxygen species (ROS). Interestingly, 1,25D and analogs have been shown to reduce both direct and indirect UV-induced DNA damage in skin cells. This was accompanied by reductions in ROS and in nitric oxide products with 1,25D following UV. Moreover, following acute UV exposure, 1,25D has been demonstrated to increase p53 levels in skin, which would presumably allow for repair of cells with damaged DNA, or apoptosis of cells with irreparably damaged DNA. Previous studies have also shown that p53 reduces intracellular ROS. Furthermore, 1,25D has been shown to induce metallothioneins, which are potent free radical scavengers. In addition to these protective effects, 1,25D has been demonstrated to inhibit stress-activated c-Jun N-terminal kinases following UV exposure, and to increase levels of the stress-induced protein heme oxygenase-1 in a model of oxidative stress. Herein, we discuss the protective effects of 1,25D and analogs in the context of UV, oxidative stress and skin cancer.

## Introduction

The worldwide incidence of both melanoma and non-melanoma skin cancers is on the rise with the main exogenous risk factor being exposure to ultraviolet (UV) radiation [1]. Solar UV radiation reaching the earth’s surface is divided into two wavebands, UVB (290–320 nm) and UVA (320-400 nm) [2], which cause both direct biological damage as well as indirect damage via the production of reactive oxygen species (ROS) such as superoxide and hydrogen peroxide [3]. Direct absorption of UVB by DNA induces the formation of DNA lesions such as cyclobutane pyrimidine dimers (CPDs) and pyrimidine (6–4) pyrimidone photoproducts [4]. In order to maintain genomic stability, the majority of such lesions are removed by DNA repair mechanisms such as nucleotide excision repair [5]. However, CPDs are considered to be more carcinogenic than the latter as they tend to be formed more often and are also less efficiently repaired [6,7]. Unrepaired DNA damage can lead to initiating mutations in skin cancer, and therefore cells with severe UV-induced DNA damage may undergo apoptosis as a protective mechanism [8].

In addition to UV-induced direct DNA damage, UV-generated ROS, or oxygen free radicals, are another mediator of apoptosis [9] and cellular senescence [10], and a major focus of this review article. UV-mediated generation of ROS involves the absorption of UV photons by photosensitisers such as riboflavin, porphyrins and quinolones [11]. Sensitisers are subsequently excited and react with oxygen to produce ROS [12]. Low levels of ROS are continuously produced *in vivo* and are involved in signal transduction pathways, cell activation, differentiation and growth control [12]. However, there is accumulating evidence of the indirect damaging effect of higher concentrations of ROS generated *in vivo* following UVA and UVB irradiation of the skin that can damage DNA, protein and lipids [12]. They also cause permanent and genetic changes involving protooncogenes and tumour suppressor genes, and activate signal transduction pathways as well as regulate genes related to growth, cellular senescence and transformation to a malignant phenotype [12]. ROS play a dual role in tumorigenesis as they can act as both an anti- and pro-tumorigenic species. They prevent tumour formation via their role in the induction of apoptosis subsequent prevention of replication of damaged cells [10]. However, by damaging cellular structures including lipids, proteins and nucleic acids, ROS inflict oxidative stress [10] and induce oxidative DNA lesions, such as 8-oxo-7,8-dihydro-2’-deoxyguanosine (8-oxodG) [13,14]. A biomarker for oxidative stress, 8-oxodG is also mutagenic and carcinogenic [15]. A redox balance is usually maintained by the cell’s antioxidant system consisting of both non-enzymatic antioxidants such as glutathione, vitamin E and retinoids, as well as antioxidant enzymes such as glutathione peroxidase, superoxide dismutase and catalase [10,16]. Despite this defence mechanism, when the formation of ROS exceeds the ability of the antioxidant system to counter them, detrimental effects such as DNA damage and lipid peroxidation may result [17].

Although exposure to UV radiation from sunlight can potentially cause photocarcinogenesis, it is also beneficial in that it constitutes our major source of vitamin D. UVB exposure converts 7-dehydrocholesterol in skin cells into pre-vitamin D_3,_ which is thermally isomerised to form vitamin D_3_ [18]. Further hydroxylation leads to the production of the active metabolite 1,25-dihydroxyvitamin D_3_ (1,25D), which can be produced in skin cells and other cell types [19,20]. Previous research in melanoma and other cancers such as colorectal carcinoma, breast carcinoma, prostate cancer, leukaemia and gastric cancer has shown an inhibitory effect of 1,25D on cancer cell viability and proliferation [21–24]. In addition, it was shown that 1,25D has an anti-proliferative effect in normal keratinocytes [25,26] while also inhibiting UV-induced apoptosis in keratinocytes, melanocytes and dermal fibroblasts [27–30]. These inhibitory effects on UV-induced apoptosis were further demonstrated to underlie a protective effect of 1,25D as it also significantly reduced UV-induced DNA damage in the form of CPDs in primary human keratinocytes, melanocytes and dermal fibroblasts [25,28–30] as well as in human subjects [31]. Such results indicate that there may be a reduced need to stimulate cellular apoptosis as there is less DNA damage. The anti-proliferative effects of 1,25D can be attributed to its effects on various regulators of the cell cycle and cell cycle pathways. Furthermore, a reduction in UV-induced 8-oxodG was observed following 1,25D treatment in keratinocytes [32], mouse skin [33] and human skin *ex vivo* [34], highlighting the protective effects of 1,25D against oxidative DNA damage. Moreover, 1,25D has been shown to reduce UV-induced ROS in primary human keratinocytes [32]. In the same recent study by Rybchyn et al., an increase in unscheduled DNA synthesis, a measure of DNA repair, was observed in UV-irradiated cells in the presence of 1,25D. Repair of DNA damage requires energy [31]. In meeting with this demand, 1,25D was shown to enhance glycolysis, thus increasing energy output for the repair of CPDs and oxidative DNA damage [32].

The most well-known physiological role of 1,25D is in the regulation of calcium and phosphate homeostasis [35]. Therefore, due to the resultant possibility of developing calcemic side effects, direct administration of pharmacological doses of 1,25D is limited [36]. Instead, low calcemic analogs of 1,25D such as 19-nor-14-epi-23-yne-1,25(OH)_2_D_3_ (TX522), 19-nor-14,20-bisepi-23-yne-1,25(OH)_2_D_3_ (TX 527) and 1α-hydroxymethyl-16-ene-24,24-difluoro-25-hydroxy-26,27-bis-homovitamin D3 (QW) have been developed which are able to replicate the photoprotective effects of 1,25D, such as the reduction in CPD formation upon UVB irradiation [25,37]. Other vitamin D analogs such as calcipotriol [38], 1α,25(OH)_2_ lumisterol_3_ (JN) and 1α,25 (OH)_2_-7-dehydrocholesterol (JM) [30] have also been reported to reduce UV-induced skin cell death. Moreover, both 1,25D and JN were demonstrated to also reduce levels of UV-induced CPDs and inhibit photocarcinogenesis in a murine model [39]. The effects of 1,25D and its analogs on UV-induced oxidative stress will be further explored here.

## 1,25D And its analogs reduce NO-mediated oxidative and nitrosative cellular damage

**Figure 1.**
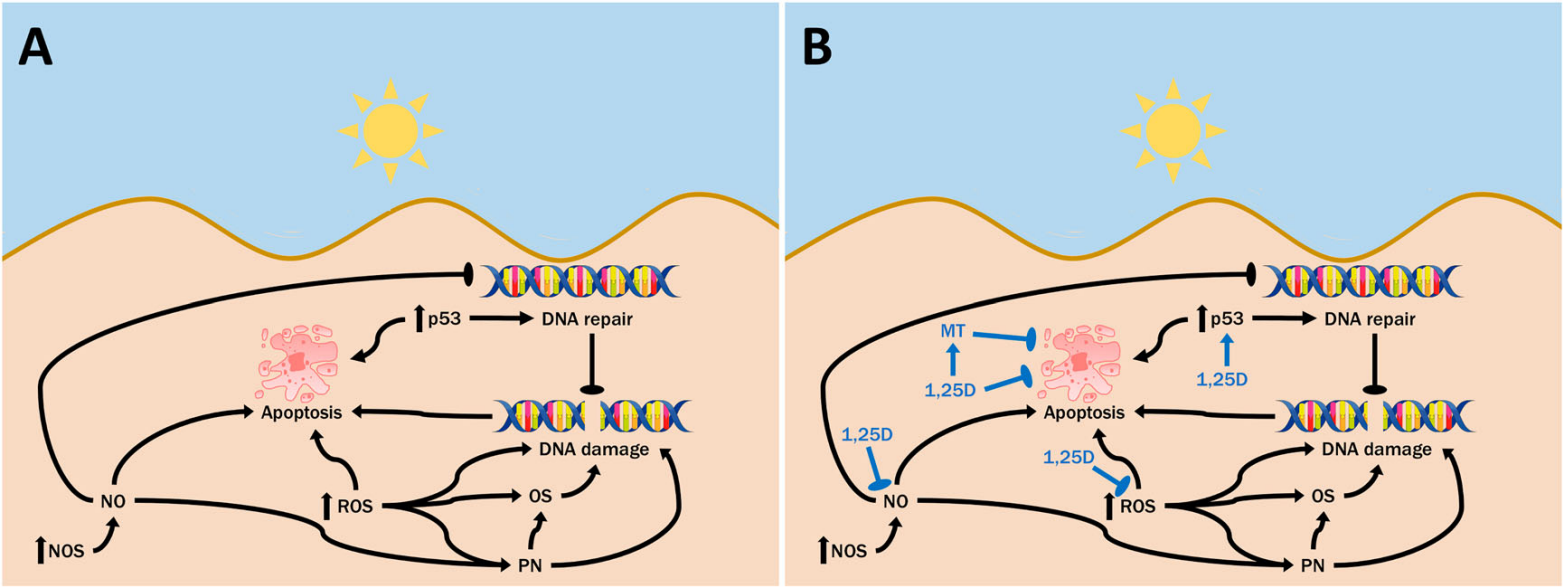
Cellular consequences of UV exposure and 1,25D. A. Ultraviolet radiation causes both direct DNA damage, as well as indirect DNA damage through UV-generated reactive oxygen species (ROS). Exposure to UV can also activate nitric oxide synthase (NOS) in the skin, increasing nitric oxide (NO) levels, which can contribute to DNA damage and also inhibit its repair. NO may combine with UV-induced superoxide to form peroxynitrite (PN), causing oxidative stress and DNA damage. UV-induced activation of p53 can facilitate DNA repair or apoptosis if the DNA is irreparably damaged, to avoid replication of cells with damaged DNA. B. 1,25D is produced in skin cells following exposure to UV. It appears to serve a photoprotective role in inhibiting levels of UV-induced DNA damage while also increasing levels of p53, which facilitates DNA repair. This is coupled with the ability of 1,25D to reduce levels of NO products as well as ROS in skin cells, which would reduce levels of indirect DNA damage and interrupt the inhibitory effect of NO on DNA repair.

The free radical nitric oxide (NO) is produced from L-Arginine by nitric oxide synthases [3]. Several studies have now confirmed that both UVA and UVB activate NOS to produce NO [11,40,41]. Therefore, the activation of nitric oxide synthase in the skin by UV increases NO levels. At high levels NO can act as a free radical or may combine with UV-induced superoxide to form the toxic reactive nitrogen species, peroxynitrite, mediating DNA damage and lipid peroxidation [3]. This is summarised in Figure 1A. Peroxynitrite-induced DNA damage can activate poly(ADP-ribose) polymerase (PARP), breaking down NAD+ into nicotinamide and ADP-ribose. This leads to a shortage of NAD+ in skin cells, reducing ATP formation and therefore energy levels, which can disrupt cell function possibly leading to cell death [42]. DNA repair will also be hindered as energy production decreases. Furthermore, peroxynitrite can also oxidise guanine leading to the production of 8-oxodG [43]. As previously mentioned, a major effect of UV radiation is the production of DNA lesions in skin cells that are removed by DNA repair enzymes. Excess levels of NO cause inactivation of such enzymes [44], inhibiting the excision and ligation steps of nucleotide excision repair [45]. Thus, it is evident that NO can impact several pathways of cellular functioning. Interestingly, NO overproduction can alter the membrane potential of mitochondria, activating the mitochondrial apoptotic pathway and allowing the release of pro-apoptotic proteins such as apoptosis initiating factor, inducing apoptosis [46]. Thus, it appears that NO has dichotomous regulatory roles and can function as both a pro- and anti-apoptotic modulator in a situation dependent fashion [47]. Studies indicate that the effect of NO depends on the cell type as well as on the stimulus that induces its production, with oxidative stress being anti-apoptotic [48]. However, long term high concentrations of NO are implicated to induce apoptotic cell death [47].

Current evidence suggests that 1,25D reduces the production of NO, thereby decreasing the incidence of oxidative and nitrosative DNA damage as well as improving DNA repair mechanisms [13], as shown in Figure 1B. It was previously demonstrated that the treatment of UV-irradiated skin cells with 1,25D significantly reduced the levels of nitrite and 3-nitrotyrosine which are used as measures for NO production [39,49]. A clear reduction in 3-nitrotyrosine was also achieved with the low calcemic 1,25D analog, JN [39]. Similarly, the treatment of human *ex vivo* skin with 1,25D also significantly reduced the level of 8-nitroguanosine, another nitric oxide product, which was markedly increased following UV irradiation [34].

## Effect of p53 on cellular redox balance and its upregulation by 1,25D

The tumour suppressor protein p53 can be induced by stresses such as UV radiation (Figure 1A), and it plays a key role in controlling the cellular consequences of DNA damage [50]. p53 activation may lead to G1 phase cell cycle arrest, allowing time for DNA damage repair before replication, and also contributes to enhanced nucleotide excision repair [49,51]. However, elevated p53 also mediates apoptosis of cells with irreparable DNA damage via the upregulation of Bax and the probable downregulation of Bcl-2 expression [49]. Studies have also identified a function for p53 in the regulation of cellular antioxidant defence mechanisms. Previous work by Sablina et al. demonstrated that p53 can decrease the levels of intracellular ROS, thereby protecting the genome from oxidative damage [52]. However, the relationship between p53 and ROS levels is complex. Conditions of severe or extended stress activate p53 leading to the expression of pro-oxidant genes, increasing ROS levels and inducing cell death. On the other hand, in response to weak to moderate stress, p53 tends to stimulate the expression of antioxidant genes that protect against cell damage [53]. Several p53 inducible antioxidant proteins have been identified such as the mitochondrial antioxidant enzyme manganese superoxide dismutase (MnSOD), which acts as the major ROS scavenging enzyme of cells. MnSOD catalyses the dismutation of superoxide decomposition into less toxic hydrogen peroxide and molecular oxygen [54]. p53 also regulates expression of glutathione peroxidase, which scavenges hydrogen peroxide, as well as other groups of antioxidant genes such as sestrins and glutaminase 2 [53].

Research in primary human skin cells showed that while p53 levels in keratinocytes [49] and melanocytes [29] were increased following UV irradiation, a more marked increase was visible in cells treated with 1,25D (1–10 nM) [29,49] and its analogs such as JN [55]. This indicates that 1,25D upregulates p53 (Figure 1B). There was also a concurrent increase in keratinocyte survival and as well as a decrease in DNA damage in the form of CPDs (thymine dimers) [49], which may be attributed to the ability of p53 to induce cell cycle arrest and improve the process of nucleotide excision repair [49]. Additionally, the resultant antioxidant effects following p53 activation may have been a contributing factor in reducing oxidative DNA damage. However, studies that compare the incidence of UV-induced oxidative DNA lesions with p53 levels upon 1,25D treatment are currently lacking but necessary to gauge a holistic understanding of the intrinsic interactions between them. A study by De Haes et al. reported a suppression of p53 accumulation upon UVB (16 mJ/cm^2^) exposure with a higher concentration of 1,25D (10 µM). This result was attributed to 1,25D’s ability to suppress UVB-induced CPD formation [25]. This reduction in CPD formation was suggested by the authors to be due to the anti-proliferative capacity of 1,25D, in that a pharmacological dose caused cell cycle arrest, rendering the DNA in these growth arrested cells more compact and in a less accessible form. It should be noted that the concentration of 1,25D used in the De Haes study was 100–1000 times higher than that used in studies by Dixon and Gupta, which used doses of 1,25D likely to be achieved in skin from sun exposure [49]. Moreover, the UV source in the De Haes study consisted of UVB (16 mJ/cm2) only whereas the UV source used in the Dixon and Gupta studies consisted of both UVB (200 mJ/cm^2^) and UVA (1170 mJ/cm^2^). These differences in 1,25D concentrations and UV irradiation procedures could well explain the discrepancy in p53 findings. Another possible explanation is the difference in culture conditions of the keratinocytes. In studies by Dixon and Gupta, the cells were cultured in the absence of EGF and cholera toxin for two days prior to irradiation to reduce overstimulation of cell signalling pathways [49], however this was not the case for the De Haes study.

## Antioxidant properties of 1,25D inducible metallothioneins

Studies by Karasawa et al. were the first to demonstrate induction of methallothionein (MT) by 1,25D in mouse keratinocytes and in mouse skin *in vivo* [56]. Indeed, the ability of 1,25D to decrease UV-induced injury of skin cells is often suggested to be mediated by MTs [57–59]. Although the physiological functions of MTs are not fully understood [60], they are potent radical scavengers, especially against hydroxyl radicals [61], and were shown to reduce hydrogen peroxide and superoxide dependent lipid peroxidation [62]. MT expression can be induced by many metals, such as cadmium (Cd), and studies have reported that Cd-induced MT can reduce sunburn cell formation *in vivo* as well as increase the survival rate of keratinocytes *in vitro* following UV irradiation [59,63]. However, due to its toxicity, Cd cannot be applied in clinical medicine. It was therefore suggested that 1,25D-induced MT may mediate a similar antioxidant effect [59]. A study conducted by Lee and Youn confirmed the photoprotective effect of 1,25D in both *in vitro* and *in vivo* systems, measured using cell viability and sunburn cell formation respectively, whilst demonstrating a concurrent increase in MT levels [59]. Sunburn cells are a histological representation of UVB mediated apoptotic keratinocytes [64]. This study also determined that the photoprotective effects of 1,25D shown in that instance were independent of the endogenous antioxidant, glutathione [59]. They suggested that there might be a switching mechanism between glutathione and MT where both will not be active at the same time and that the effects shown in Lee and Youn’s study are due to MT and not glutathione which is another potent antioxidant. In addition, Karasawa et al demonstrated that 1,25D induced MT mRNA in epidermal keratinocytes *in vitro* and in liver, kidney and skin tissues *in vivo* [56]. The photoprotective effect of MT was further confirmed by the fact that MT-null mice had a greater number of sunburn cells in their skin after UV irradiation compared to normal mice [63], thus demonstrating reduced tolerance in MT-null mice against UV-induced injury to the skin.

Owing to side chain modifications, the low calcemic 1,25D analogs 19-Nor-14-epi-23-yne-1,25 dihydroxyvitamin D3 (TX522) and 1α,25(OH)2-19-nor-14,20-bisepi-23-yne-vitamin D3 (TX527) have enhanced antiproliferative and pro-differentiating capacity, both being at least ten times more potent than 1,25D in inhibiting primary human keratinocyte proliferation [58]. Both analogs were also reported to induce MT [25] with this therefore being a likely contributing mechanism for the antioxidant effects of 1,25D, TX522 and TX527. Similar to the action of 1,25D, TX522 and TX527 also reduced UVB-induced apoptosis of primary human keratinocytes by suppressing PARP cleavage [25]. Since apoptosis is a protective mechanism to remove cells with irreparable DNA damage, its suppression may not necessarily indicate increased skin cancer risk, but may actually be a result of reduced oxidative damage, attributable to the action of MT.

## Inhibition of the stress activated c-Jun N-terminal kinases by 1,25D

The mitogen activated protein kinases (MAPKs) are a group of serine/threonine kinases that phosphorylate and sequentially activate one another in response to stimuli involved in the regulation of a variety of cellular processes such as proliferation, differentiation and apoptosis [50]. The c-Jun N-terminal kinases (JNKs) are a subfamily of the MAPKs, and are also referred to as stress activated protein kinases due to their role in the mediation of the stress induced cellular response [65]. Several studies have reported strong activation of JNKs in keratinocytes by UV, with further evidence indicating the involvement of ROS in this process [66]. Activation of the JNK signalling cascade by stress stimuli has been associated with the induction of cellular apoptosis as a protective mechanism against photocarcinogenesis [65]. Previous work using fibroblasts demonstrated that JNK is involved in UV-induced apoptosis via the release of cytochrome c from mitochondria [67]. It was also shown that the pretreatment of normal human keratinocytes with 1,25D lead to a 30–50% reduction in the UVB-induced phosphorylation of JNK. Furthermore, while UVB irradiation promoted cytochrome c release in keratinocytes, this effect was suppressed by 1,25D. These anti-apoptotic effects could well be explained by the reductions in UV-induced cellular damage with 1,25D treatment, resulting in reduced need for eradication of irreparably damaged cells [27].

## Role for heme oxygenase-1 in the photoprotective effects of 1,25D

The enzyme heme oxygenase (HO) catalyses the rate limiting step in the degradation of cellular heme yielding carbon monoxide, biliverdin and free iron (Fe^2+^) [68]. Three isoforms of this enzyme have been identified with HO-1 being the protein that is inducible by various stimuli, such as UV radiation [69]. Studies in bacterial cells have shown that UVA irradiation of heme-containing proteins can produce singlet oxygen resulting in oxidative damage [70], therefore a transient reduction in heme levels by HO-1 induction may serve a cytoprotective purpose.

Fe^2+^ released following HO-1 action is rapidly sequestered into the iron storage protein, ferritin [69]. This process prevents Fe^2+^ from participating in the ROS generating Fenton reaction [68]. In addition, it has been shown that ferritin levels increase following oxidative stress [71], further enhancing the protective effect. On the other hand, biliverdin generated by the breakdown of heme is subsequently converted into bilirubin by biliverdin reductase with both biliverdin and bilirubin being powerful antioxidants, further contributing to the antioxidative effect of HO-1 [68].

Oerman et al. examined the effect of 1,25D on HO-1 expression in glial cells of photothrombotically lesioned rats. The photothrombosis model was used to elicit focal cortical ischemia, resulting in oxidative stress and the induction of HO-1 expression. Post-lesional treatment with 1,25D led to a transient but significant further increase in HO-1 expression [72]. A similar response to 1,25D may be obtained in skin cells following UV irradiation, however few studies have been conducted in this area and further work is required to confirm this.

## 1,25D And its analogs may induce antioxidant genes

The above sections discussed some of the probable mechanisms by which 1,25D may indirectly upregulate antioxidant genes. Gene expression profiling techniques were utilised to discover that 1,25D and analogs can induce several antioxidant genes such as glucose-6-phosphate dehydrogenase, glutathione peroxidase and thioredoxin reductase [73]. Research conducted on non-malignant human prostate epithelial cell lines showed that 1,25D offered protection against hydrogen peroxide-induced cell death [73]. It was also found that 1,25D pre-treatment protected against a hydrogen peroxide challenge by promoting glucose-6-phosphate dehydrogenase activity, which in turn increased glutathione levels [73]. Therefore, ROS levels were decreased, reducing oxidative cellular injuries. However, in cancer cells, 1,25D appears to undergo a role reversal, exerting pro-oxidative effects. It has been shown to exacerbate TNF-induced depletion of the glutathione pool [74] and reduce zinc superoxide dismutase activity in cancer cells [75], thus decreasing the antioxidant capacity. This effect of 1,25D in transformed cells may be useful in increasing cancer cell susceptibility to cytotoxic agents, thus holding therapeutic potential.

## Conclusion

An inhibitory effect of 1,25D against cancers such as melanoma, breast and gastric cancer has previously been identified. In addition to this, and as shown in Figure 1, extensive evidence demonstrates that 1,25D can also act as a photoprotective agent, reducing UV-induced DNA damage. The decrease in 8-oxodG following 1,25D treatment is an example of this. Some of the possible mechanisms behind the effects of 1,25D against oxidative stress have been discussed here with the induction of MT believed to play a major role. Better identification of the manner in which 1,25D exerts its photoprotective effects may allow the development of targeted approaches to both prevent and treat skin cancer.
